# Diagnostic Value of Circulating Long Non-Coding RNAs *HOTAIR*, *NEAT1*, and *CCAT1* for Colorectal Cancer: A Vietnamese Case–Control Study

**DOI:** 10.3390/cimb48050433

**Published:** 2026-04-22

**Authors:** Khanh Ngoc Nguyen, Diem Thi Nguyen, Khanh Hong Pham, Chau Pham, Huy Quang Duong, Thuy Thi Bich Vo

**Affiliations:** 1 Military Hospital 103, Vietnam Military Medical University, 261 Phung Hung, Ha Dong, Hanoi 100000, Vietnam; dr.khanh.v103@gmail.com (K.N.N.); drpham103@gmail.com (K.H.P.); bsphamchau2011@gmail.com (C.P.); 2 Institute of Biology, Vietnam Academy of Science and Technology, 18 Hoang Quoc Viet, Nghia Do, Hanoi 100000, Vietnam; diemly0927@gmail.com

**Keywords:** colorectal cancer, long non-coding RNA, *CCAT1*, *HOTAIR*, *NEAT1*, liquid biopsy

## Abstract

Circulating long non-coding RNAs (lncRNAs) have emerged as promising non-invasive biomarkers for colorectal cancer (CRC) detection; however, data in Vietnamese populations remain limited. In this study, a total of 218 participants (106 CRC, 80 adenomas, and 32 healthy controls) were included. Relative expression levels and diagnostic performance of three circulating lncRNAs—*CCAT1*, *HOTAIR*, and *NEAT1*—were quantified using RT-qPCR and analyzed by the 2^−ΔΔCt^ method. Receiver operating characteristic (ROC) curve analysis was performed to assess the diagnostic accuracy of individual lncRNAs and their combinations. *CCAT1*, *HOTAIR*, and *NEAT1* were significantly upregulated in CRC patients compared with adenoma patients and healthy controls (all *p* < 0.001). Expression levels were higher in advanced-stage (TNM III–IV) CRC than in early-stage disease. Among individual markers, *HOTAIR* demonstrated the highest diagnostic accuracy (AUC = 0.918), followed by *CCAT1* (AUC = 0.908) and *NEAT1* (AUC = 0.890). Combined biomarker models showed improved performance, with the *CCAT1* + *HOTAIR* combination achieving the highest AUC (0.944). Overall, circulating *CCAT1*, *HOTAIR*, and *NEAT1* demonstrated favorable diagnostic performance in a Vietnamese population and outperformed conventional markers (CEA and CA19-9). These findings support the potential utility of multi-lncRNA panels as non-invasive biomarkers for CRC detection, warranting further validation in larger, independent cohorts.

## 1. Introduction

Colorectal cancer is a major global health concern and one of the most lethal malignancies worldwide. According to GLOBOCAN 2022, CRC ranks as the third most commonly diagnosed cancer, with an estimated 1.93 million new cases, and stands as the second leading cause of cancer-related mortality, accounting for approximately 904,859 deaths annually [1]. Despite the increasing availability of treatment options, the prognosis of CRC largely depends on the stage at diagnosis. Early-stage detection dramatically improves survival rates, with five-year survival reaching over 90%, compared to less than 15% in metastatic cases [2]. However, due to the often silent nature of early CRC and limited screening coverage, many patients are diagnosed at advanced stages.

In Vietnam, CRC is among the five most common cancers. Based on GLOBOCAN 2022 data, there are an estimated 16,835 new cases and 8454 deaths each year. Importantly, a large proportion of patients are diagnosed at late stages, with 67.8% of cases detected at stages III–IV, underscoring significant shortcomings in current screening strategies within the Vietnamese healthcare system [3].

Currently, colonoscopy remains the gold standard for CRC screening and diagnosis. While highly specific and sensitive, this method is invasive, costly, and not widely accessible, especially in low- and middle-income countries like Vietnam [4]. Consequently, there is a pressing need for alternative, non-invasive, and reliable biomarkers that can facilitate early detection and improve patient outcomes [5]. In this context, there is a need to explore alternative approaches that are feasible within resource-limited settings while maintaining acceptable diagnostic performance.

In recent years, long non-coding RNAs have emerged as a novel class of regulatory RNAs with key roles in cancer biology [6]. LncRNAs are defined as RNA transcripts longer than 200 nucleotides that lack canonical open reading frames sufficient for encoding functional proteins; however, emerging evidence suggests that a small subset may encode short functional peptides, indicating that their protein-coding capacity is not entirely absent but remains highly limited and context-dependent [7]. These molecules are involved in diverse cellular processes including epigenetic regulation, chromatin remodeling, transcriptional control, and post-transcriptional modification [8,9]. Despite their biological importance, lncRNAs remain challenging to study due to their typically low expression levels, high tissue specificity, poor sequence conservation across species, and context-dependent regulatory functions [10]. In this study, these challenges were partially addressed by focusing on well-characterized lncRNAs with reproducible detection in peripheral blood and by applying sensitive quantitative real-time PCR methods to ensure reliable measurement.

Among the many lncRNAs implicated in CRC, *CCAT1* (Colon Cancer-Associated Transcript 1), *HOTAIR* (HOX Transcript Antisense Intergenic RNA), and *NEAT1* (Nuclear-Enriched Abundant Transcript 1) have been consistently reported to contribute to tumorigenesis, progression, and metastasis. *CCAT1* is known to promote cell proliferation and invasion via MYC signaling and is overexpressed in CRC tissues and plasma [11]. *HOTAIR* is associated with poor prognosis and has been shown to regulate epithelial–mesenchymal transition (EMT), enhancing tumor aggressiveness [12]. *NEAT1*, involved in paraspeckle formation and gene regulation, has been linked to tumor progression, chemoresistance, and immune escape in CRC [13]. Notably, several studies have demonstrated that *HOTAIR* and *NEAT1* exhibit high diagnostic accuracy for CRC when measured in peripheral blood, suggesting their utility in non-invasive screening [14,15].

In the Vietnamese context, several environmental and lifestyle factors may influence lncRNA expression and thereby affect their potential as biomarkers. These include a diet increasingly rich in red and processed meats and low in dietary fiber, a high prevalence of tobacco uses among males, widespread alcohol consumption, and exposure to foodborne contaminants such as nitrosamines and pesticide residues. In addition, chronic gastrointestinal infections and inflammation, along with alterations in gut microbiota associated with rapid urbanization, may further modulate gene expression patterns [16]. Mechanistically, these factors can contribute to epigenetic dysregulation, including aberrant DNA methylation, histone modification, and oxidative stress, which in turn may influence the expression of lncRNAs such as *CCAT1*, *HOTAIR*, and *NEAT1*.

While the roles of these lncRNAs in CRC have been extensively explored in other populations, evidence remains limited to specific geographic and ethnic cohorts, and cross-population generalizability has not been fully established. To date, there has been no published study investigating their expression or diagnostic value in a Vietnamese population, highlighting a critical gap in geographically representative data.

To address this gap, we conducted a study to evaluate the expression levels of *CCAT1*, *HOTAIR*, and *NEAT1* in the peripheral blood of Vietnamese patients diagnosed with CRC, in comparison to patients with colorectal adenomas. Furthermore, we assessed the diagnostic performance of each lncRNA individually and in combination, to determine their potential clinical utility. By generating data from a clearly defined geographic population, this study aims to provide context-specific evidence and contribute to a more nuanced understanding of lncRNA-based biomarkers in CRC.

## 2. Materials and Methods

### 2.1. Study Population

This study was conducted at the 103 Military Hospital in collaboration with the Vietnam Academy of Science and Technology from October 2023 to October 2024. A total of 218 participants were enrolled and categorized into two main groups: CRC group consisting of 106 patients newly diagnosed with CRC, and a control group comprising 112 individuals, including 80 patients with histologically confirmed colorectal adenomas (adenomatous polyps) and 32 healthy individuals with no evidence of malignancy or colorectal disease.

Exclusion criteria included patients who had received prior treatment for CRC; those with secondary colorectal malignancies or other concurrent malignancies; patients with coexisting inflammatory bowel diseases such as ulcerative colitis or Crohn’s disease; individuals under 18 years of age; and those who did not provide informed consent.

Sample size was estimated based on ROC analysis using a hypothesis-testing approach for comparing the expected area under the curve (AUC) with the null hypothesis (AUC = 0.5). The calculation was performed using the following formula:

$$n=\frac{{\left(Z_{\alpha }+Z_{\beta }\right)}^{2}(V_{1}+V_{0})}{({{AUC}_{1}-{AUC}_{0})}^{2}}$$

where *AUC*_1_ represents the expected AUC, *AUC*_0_ is the null hypothesis value (0.5), *V*_1_ and *V*_0_ denote the variance of the AUC in the diseased and non-diseased groups, respectively, and *Zα* and *Zβ* correspond to the desired significance level and statistical power.

Based on previous studies reporting AUC values ranging from 0.78 to 0.91, an expected AUC of 0.83 was assumed. With α = 0.05 and 80% power, the minimum required sample size was estimated to be 73 subjects per group. To improve model robustness and reduce potential overfitting, the sample size was increased to at least 100 subjects per group.

### 2.2. Study Design and Sample Selection

This was a descriptive cross-sectional study with a control group. Participants were selected by convenience sampling, based on the inclusion and exclusion criteria. Clinical and demographic data, including age, sex, presenting symptoms, tumor location (via colonoscopy), tumor size, and disease stage (based on abdominal CT scans and Tumor–Node–Metastasis (TNM) system), were collected using standardized forms.

Disease staging was classified according to the TNM system of the American Joint Committee on Cancer (AJCC Cancer Staging Manual, 8th edition, 2017).

Serum tumor markers, including carcinoembryonic antigen (CEA) and carbohydrate antigen 19-9 (CA19-9), were also collected as part of routine clinical evaluation. CEA levels were quantified using enzyme-linked immunosorbent assay (ELISA) and electrochemiluminescence immunoassay methods, as performed routinely in hospital laboratories, with a normal reference range of 0–5 ng/mL. CA19-9 levels were measured using electrochemiluminescence immunoassay, according to standard clinical protocols.

### 2.3. RNA Extraction and Quantification of lncRNA Expression

Expression levels of three lncRNAs (*CCAT1*, *HOTAIR*, and *NEAT1*) were assessed using qPCR in peripheral blood samples from a subset of 218 selected (106 CRC, 80 adenomas controls, and 32 healthy controls).

Primer sequences used in this study were verified against the latest NCBI GenBank database using BLAST (version 2.17.0) to ensure specificity and to avoid off-target amplification.

Sample preparation: A total of 2 mL of peripheral blood was collected in EDTA tubes. Samples were centrifuged at approximately 447× *g* for 10 min to reduce red blood cell content and obtain a leukocyte-enriched fraction, rather than isolating cell-free plasma. The processed blood fraction was retained for downstream RNA extraction and stored at −80 °C until further processing.

RNA isolation and cDNA synthesis: Total RNA was extracted from the processed whole blood fraction (including cellular components) using TRIzol reagent (Invitrogen, Carlsbad, CA, USA) according to the manufacturer’s protocol. RNA quantity and purity were assessed using a NanoDrop spectrophotometer (Thermo Fisher Scientific, Waltham, MA, USA). Only samples with an OD260/280 ratio between 1.8 and 2.0 were used. Complementary DNA (cDNA) was synthesized from 0.5 μg of total RNA using Maxime RT PreMix Kit (Intron Biotechnology, Seongnam, Republic of Korea).

Real-time PCR (qPCR): Gene expression levels of *CCAT1*, *HOTAIR*, *NEAT1*, and *GAPDH* (reference gene) were quantified using SYBR Green-based RT-qPCR (RealMOD™ Green W2 2X qPCR Mix, Intron Biotechnology, Seongnam, Republic of Korea). Each 11 µL reaction contained 5.5 µL of qPCR mix, 0.55 µL of each forward and reverse primer (Table 1), 1.1 µL of cDNA template, and 3.3 µL of DEPC-treated water. The amplification protocol included initial denaturation at 95 °C for 20 s, followed by 40 cycles of annealing (gene-specific temperature) for 40 s, and extension at 72 °C for 30 s.

### 2.4. Statistical Analysis

All data were processed and analyzed using R software (version 4.4.3, R Foundation for Statistical Computing, Vienna, Austria). Threshold cycle (Ct) values of target genes were obtained from qPCR assays, with *GAPDH* used as the endogenous reference gene for normalization. The ΔCt value was calculated as follows: ΔCt = Ct_target − Ct_GAPDH. The ΔΔCt value was then determined by subtracting the mean ΔCt of the healthy control group from the ΔCt of each study sample (ΔΔCt = ΔCt_sample − mean ΔCt_control). Relative gene expression levels were calculated using the 2^−ΔΔCt^ method to compare expression differences among study groups [17]. These 2^−ΔΔCt^ values represent relative fold changes in gene expression normalized to GAPDH, and calibrated against the healthy control group.

For quantitative analyses, including receiver operating characteristic curve analysis, determination of optimal cut-off values, calculation of sensitivity, specificity, positive predictive value, negative predictive value, and *p*-values, relative expression data expressed as 2^−ΔCt^ were used [18]. The non-cancer control group included patients with adenoma and healthy individuals, allowing assessment of the diagnostic performance of the selected lncRNAs in distinguishing CRC from both precancerous and normal conditions.

All statistical analyses and data visualization were performed in the R environment using the dplyr, ggplot2, pROC, and writexl packages. All statistical tests were two-tailed, and a *p*-value < 0.05 was considered statistically significant.

Receiver operating characteristic (ROC) curve analysis was performed to evaluate the diagnostic performance of each biomarker. The optimal cut-off values were determined using Youden’s Index (J = sensitivity + specificity − 1), which identifies the point that maximizes the combined sensitivity and specificity.

## 3. Results

### 3.1. Participant Characteristics

The general characteristics of the study participants are summarized in Table 2. The mean age did not differ significantly among the CRC, adenoma, and healthy groups (Kruskal–Wallis test, *p* = 0.079). Similarly, no significant difference in gender distribution was observed across the study groups (Chi-square test, *p* = 0.165), although males predominated in the overall study population. These findings indicate that the study groups were comparable in terms of basic demographic characteristics, minimizing potential confounding effects of age and sex on subsequent analyses.

### 3.2. Tumor Characteristics of Colorectal Cancer Patients

Tumor characteristics of CRC patients are summarized in Table 3. Tumors were most frequently located in the rectum (40.6%), followed by the left colon (38.7%) and right colon (20.7%). The majority of tumors involved ≥¾ of the bowel circumference (76.4%), whereas smaller tumors were less common. According to TNM staging, most patients were diagnosed at advanced stages, with stage III (35.8%) and stage IV (26.4%) accounting for more than half of the cases, while early-stage disease (stages I–II) represented 37.8% of the cohort.

### 3.3. Expression Levels of CCAT1, HOTAIR, and NEAT1

Relative expression levels were calculated using the 2^−ΔΔCt^ method. Horizontal lines indicate median values. Statistical comparisons were performed using pairwise Wilcoxon rank-sum tests followed by post hoc pairwise comparisons. *p*-values are shown in the figure.

The median expression level of *CCAT1* in CRC patients was 13.47 (IQR: 6.12–28.17), which was markedly higher than that in the adenoma group [2.30 (0.52–3.81)] and healthy controls [1.00 (0.41–2.37)]. Similarly, *NEAT1* showed a median expression of 13.73 (5.75–40.66) in the CRC group, compared with 1.96 (0.47–4.04) in adenoma patients and 1.00 (0.29–1.77) in healthy individuals. For *HOTAIR*, CRC patients also exhibited elevated expression, with a median value of 8.52 (4.60–24.66), whereas expression levels in the adenoma [1.14 (0.45–2.26)] and healthy [1.01 (0.46–1.53)] groups were comparable.

**Figure 1:**
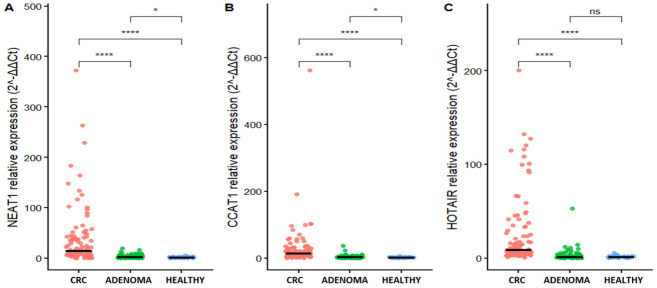
Relative expression levels of lncRNAs *NEAT1* (**A**), *CCAT1* (**B**), and *HOTAIR* (**C**) among colorectal cancer, adenoma, and healthy groups. Statistical comparisons were performed using the Wilcoxon rank-sum test. * *p* < 0.05; **** *p* < 0.0001, while “ns” indicates no statistically significant difference.

As shown in Figure 1, the relative expression levels of *CCAT1*, *NEAT1*, and *HOTAIR* differed significantly among the CRC, adenoma, and healthy groups. *CCAT1* and *NEAT1* exhibited a clear gradient of increasing expression from healthy controls to adenoma patients and further to CRC patients, with the highest levels observed in the CRC group (all pairwise comparisons, *p* < 0.05). In contrast, *HOTAIR* expression was significantly elevated in CRC compared with both adenoma patients and healthy controls (*p* < 0.001), whereas no statistically significant difference was observed between the adenoma and healthy groups (*p* = 0.34).

### 3.4. Association Between lncRNA Expression Levels and TNM Stage in Colorectal Cancer

**Figure 2:**
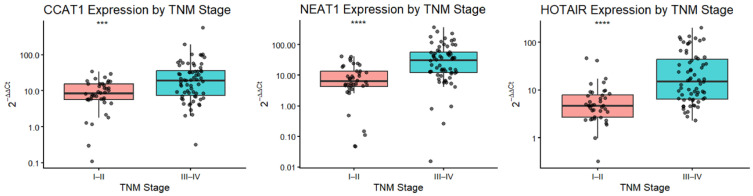
Relative expression levels of *CCAT1*, *NEAT1*, and *HOTAIR* according to TNM stage in colorectal cancer patients. Patients were grouped into early-stage (TNM I–II) and advanced-stage disease (TNM III–IV). Relative expression levels were calculated using the 2^−ΔΔCt^ method. Data are presented as boxplots showing the median and interquartile range, with individual data points overlaid. Statistical comparisons were performed using the Wilcoxon rank-sum test. *** *p* < 0.001; **** *p* < 0.0001.

When stratified by tumor stage, the relative expression levels of *CCAT1*, *HOTAIR*, and *NEAT1* were significantly higher in patients with advanced CRC (TNM stages III–IV) compared with those with early-stage disease (stages I–II) (Figure 2). Specifically, *CCAT1* expression was significantly increased in stages III–IV relative to stages I–II (*p* < 0.001). Similarly, *NEAT1* and *HOTAIR* showed markedly higher expression levels in advanced-stage CRC, with highly significant differences between the two stage groups (*p* < 0.0001 for both). These findings indicate that elevated expression of *CCAT1*, *NEAT1*, and *HOTAIR* is associated with more advanced tumor stage, suggesting a potential link between these lncRNAs and CRC progression.

### 3.5. Diagnostic Performance of Individual and Combined lncRNAs

To further evaluate the diagnostic utility of *CCAT1*, *HOTAIR*, and *NEAT1*, ROC curve analysis was performed to assess the discriminative ability of each individual lncRNA and their combinations in distinguishing CRC patients from non-cancer controls (adenoma and healthy groups).

The diagnostic performance of *CCAT1*, *NEAT1*, *HOTAIR*, and their combined models for CRC is summarized in Table 4. Among individual biomarkers, *HOTAIR* achieved the highest diagnostic accuracy, with an area under the ROC curve (AUC) of 0.918 (95% CI: 0.823–0.922), a sensitivity of 87.7%, and a specificity of 84.8%. *CCAT1* and *NEAT1* also demonstrated good discriminative ability, with AUC values of 0.908 (95% CI: 0.841–0.935) and 0.890 (95% CI: 0.842–0.935), respectively (all *p* < 0.0001).

Notably, combined biomarker models showed improved diagnostic performance compared with individual lncRNAs. The combination of *CCAT1* and *HOTAIR* yielded the highest AUC (0.944), with a sensitivity of 94.3% and a specificity of 85.7%. Similarly, the *HOTAIR* + *NEAT1* model achieved an AUC of 0.940, while the *CCAT1* + *NEAT1* combination showed the highest specificity (95.5%) among all models. These results indicate that combining lncRNAs enhances the diagnostic accuracy for CRC compared with single-marker approaches.

**Figure 3:**
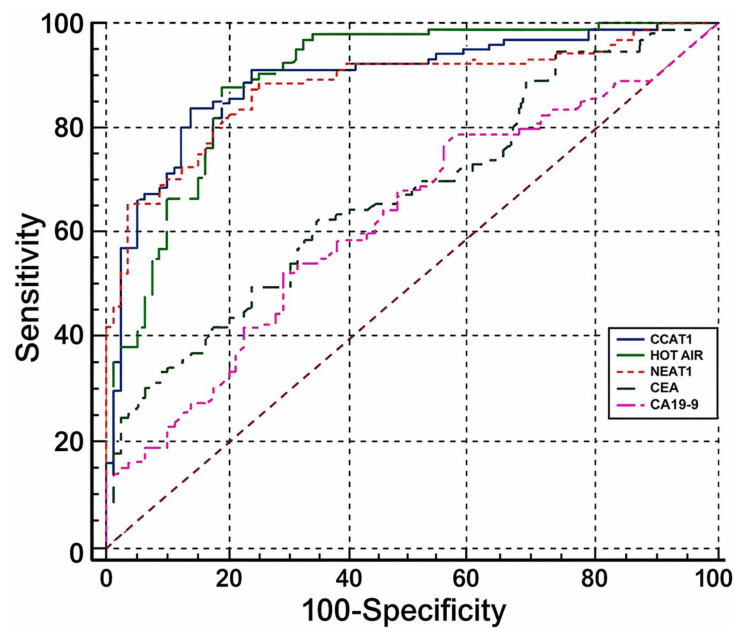
Receiver operating characteristic (ROC) curves of *CCAT1*, *HOTAIR*, *NEAT1*, CEA and CA19-9 for distinguishing CRC patients from non-cancer controls.

In addition, the diagnostic performance of the investigated lncRNAs was compared with conventional serum tumor markers, including CEA and CA19-9. The AUC values for CEA and CA19-9 were 0.675 (95% CI: 0.602–0.742) and 0.627 (95% CI: 0.553–0.697), respectively, which were markedly lower than those of all three lncRNAs. Statistical comparisons demonstrated that *CCAT1*, *HOTAIR*, and *NEAT1* significantly outperformed both CEA and CA19-9 (all *p* < 0.001). These findings highlight the superior diagnostic value of circulating lncRNAs compared with conventional biomarkers (Figure 3).

## 4. Discussion

In the present study, we investigated the expression patterns and diagnostic performance of three circulating lncRNAs—*CCAT1*, *HOTAIR*, and *NEAT1*—in patients with CRC compared with individuals with colorectal adenomas and healthy controls. Our results showed that all three lncRNAs were significantly upregulated in CRC patients and exhibited favorable diagnostic performance, both individually and in combination, suggesting their potential relevance as non-invasive biomarkers for CRC detection.

Among the investigated lncRNAs, *HOTAIR* exhibited markedly elevated expression levels in CRC patients, with a median value of 8.52, which was significantly higher than that observed in the adenoma group (median 1.14, *p* < 0.001). *HOTAIR* also demonstrated excellent diagnostic performance, with an AUC of 0.918; at the optimal cut-off value of 2.22, it achieved a sensitivity of 87.7% and a specificity of 84.8%. These findings are highly consistent with previous studies. Ismail et al. (2019) [12] reported that circulating *HOTAIR* expression was increased by approximately 7.55-fold in CRC patients compared with healthy controls and showed strong diagnostic accuracy. In addition, Chen et al. (2020) [14] confirmed that *HOTAIR* is highly expressed in CRC tissues and may play an important role in promoting tumor metastasis. Together, these data support the association between *HOTAIR* upregulation and CRC development.

*NEAT1* has also been widely reported to be overexpressed in CRC. Wu et al. (2015) [13] demonstrated that circulating *NEAT1* levels were significantly higher in CRC patients than in healthy individuals, and that two *NEAT1* transcript variants (*NEAT1*-v1 and *NEAT1*-v2) showed good diagnostic accuracy, with AUC values of 0.732 and 0.845, respectively. Consistent with these findings, our study showed that *NEAT1* expression was substantially higher in CRC patients (median 13.73) than in adenoma patients (median 1.96), suggesting a possible association between *NEAT1* upregulation and disease progression. Furthermore, *NEAT1* demonstrated good diagnostic performance in distinguishing CRC from adenoma and healthy groups, with an AUC of 0.890, sensitivity of 87.7%, and specificity of 81.2%. *NEAT1* has been implicated in multiple oncogenic processes, including tumor proliferation, metastasis, chemoresistance, and regulation of the tumor immune microenvironment, which may partially explain its elevated expression in CRC.

Similarly, *CCAT1* was significantly upregulated in CRC patients in our cohort, with the highest median expression level observed in the CRC group (13.47) compared with the adenoma group (2.30). *CCAT1* also showed strong diagnostic performance, with an AUC of 0.908. These findings are in line with previous studies reporting elevated *CCAT1* expression in CRC and its association with disease progression. Gu et al. (2020) [15] demonstrated that *CCAT1* was highly expressed in CRC tissues and closely related to tumor progression. The observed stepwise increase in *CCAT1* expression from healthy controls to adenoma patients and further to CRC patients suggests a possible involvement of *CCAT1* in the adenoma–carcinoma sequence.

Importantly, when compared with conventional serum tumor markers such as CEA and CA19-9, all three lncRNAs demonstrated superior diagnostic performance. The AUC values of CEA (0.675) and CA19-9 (0.627) were markedly lower than those observed for *CCAT1*, *HOTAIR*, and *NEAT1*. These findings are in line with previous studies indicating that circulating lncRNAs may provide higher sensitivity and specificity than traditional protein biomarkers in CRC detection [19].

The improved diagnostic performance observed with combined lncRNA models is consistent with previous reports demonstrating synergistic effects between *CCAT1* and *HOTAIR* [11]. However, rather than representing a conceptual breakthrough, our findings primarily serve as an external validation of previously reported biomarker combinations in a Vietnamese population. This highlights the importance of population-specific validation, as genetic background, environmental exposures, and lifestyle factors may influence biomarker expression and diagnostic performance.

From a practical perspective, the implementation of RT-qPCR-based multi-marker panels in routine clinical practice in Vietnam may face several challenges. These include limited laboratory infrastructure in lower-level healthcare settings, variability in pre-analytical sample handling, lack of standardized protocols for circulating RNA analysis, and cost-related constraints associated with multiplex testing. Similar barriers have been reported in other low- and middle-income countries, where access to advanced molecular diagnostics remains limited [5]. Therefore, further efforts are required to standardize methodologies and evaluate the feasibility and cost-effectiveness of these assays in real-world clinical settings.

Although we observed clear differences in lncRNA expression between CRC and non-cancer groups, and potential associations with disease stage, this study did not include longitudinal follow-up or survival analysis. Therefore, the prognostic value of these biomarkers, including their ability to predict overall survival or disease progression, could not be assessed and remains to be clarified in future studies.

Overall, our findings suggest that circulating *CCAT1*, *HOTAIR*, and *NEAT1* are promising diagnostic biomarkers for CRC, particularly when used in combination.

This study has several limitations. First, the sample size was relatively modest and derived from a single center, which may limit the generalizability of the findings. Second, the cross-sectional design precludes assessment of temporal changes and causal relationships. Third, external validation in independent cohorts was not performed. Finally, the use of peripheral blood-derived RNA rather than strictly cell-free RNA may introduce variability when comparing results with plasma-based studies.

Future studies with larger, multi-center cohorts, standardized analytical protocols, and longitudinal follow-up are warranted to validate these findings, assess prognostic value, and further explore the clinical applicability of circulating lncRNA-based biomarker panels for CRC detection.

## 5. Conclusions

In conclusion, *CCAT1*, *HOTAIR*, and *NEAT1* were significantly upregulated in CRC and demonstrated good diagnostic performance, particularly when combined. These lncRNAs outperformed conventional markers (CEA and CA19-9), supporting their potential as non-invasive biomarkers in a Vietnamese population. However, further validation in larger cohorts is required before clinical application.

## Figures and Tables

**Table 1:** Primer sequences used in this study.

Gene	Primer Sequence (5′–3′)	Annealing Temp (°C)	Product Size (bp)
*CCAT1*	F: CATTGGGAAAGGTGCCGAGA	60	139
	R: ACGCTTAGCCATACAGAGCC		
*HOTAIR*	F: AATAGACATAGGAGAACACTT	55	155
	R: AATCTTAATAGCAGGAGGAA		
*NEAT1*	F: CAGAGACACAGGCATTCA	53	130
	R: GACTACACTCCTTGGTAACT		
*GAPDH*	F: ATGGTGAAGGTCGGTGTGA	55	129
	R: CCATGTAGTTGAGGTCAATGAG		

**Table 2:** General characteristics of study participants.

Characteristics		Total (*n* = 218)	CRC Group (*n* = 106)	Adenomas Group (*n* = 80)	Healthy Group (*n* = 32)	*p* Value
Age (mean ± SD)		64.8 ± 12.6	66.1 ± 13.3	63.6 ± 10.9	63.8 ± 13.9	0.079
Gender	Male (*n*, %)	131 (60.1)	63 (59.4)	53 (66.2)	15 (46.9)	0.165
	Female (*n*, %)	87 (39.9)	43 (40.6)	27 (33.8)	17 (53.1)	

**Table 3:** Tumor characteristics in CRC patients (*n* = 106).

Feature Tumor Location	Number of Patients	Percentage (%)
Right colon	22	20.7
Left colon	41	38.7
Rectum	43	40.6
Tumor Size		
≤¼ circumference	6	5.7
¼–≤½ circumference	6	5.7
½–≤¾ circumference	13	12.3
≥¾ circumference	81	76.4
Tumor Stage (TNM)		
I	11	10.4
II	29	27.4
III	38	35.8
IV	28	26.4

**Table 4:** Diagnostic performance of lncRNAs and combinations.

Marker(s)	Cut-Off	AUC	Sensitivity (%)	Specificity (%)	PPV (%)	NPV (%)	*p*-Value
*CCAT1*	1.178	0.908	90.6	82.1	82.8	90.2	<0.0001
*NEAT1*	1.639	0.890	87.7	81.2	81.6	87.5	<0.0001
*HOTAIR*	2.22	0.918	87.7	84.8	84.5	88.0	<0.0001
*CCAT1* + *HOTAIR*	-	0.944	94.3	85.7	86.2	94.1	<0.0001
*HOTAIR* + *NEAT1*	-	0.940	90.6	83.9	84.2	90.4	<0.0001
*CCAT1* + *NEAT1*	-	0.926	80.2	95.5	94.4	83.6	<0.0001

## Data Availability

The original contributions presented in this study are included in the article. Further inquiries can be directed to the corresponding author.

## Abbreviations

The following abbreviations are used in this manuscript:

CRC	Colorectal cancer
lncRNAs	long non-coding RNAs
ROC	Receiver operating characteristic
*CCAT1*	Colon Cancer-Associated Transcript 1
*HOTAIR*	HOX Transcript Antisense Intergenic RNA
*NEAT1*	Nuclear-Enriched Abundant Transcript 1
cDNA	Complementary DNA
Ct	Threshold cycle
TNM	Tumor Stage
